# Straight to the point: targeted mRNA-delivery to immune cells for improved vaccine design

**DOI:** 10.3389/fimmu.2023.1294929

**Published:** 2023-11-27

**Authors:** Bruna Clemente, Maxime Denis, Camila Pedroso Silveira, Francesca Schiavetti, Michela Brazzoli, Daniela Stranges

**Affiliations:** GSK, Siena, Italy

**Keywords:** mRNA vaccine, dendritic cells, targeted delivery, C-type lectins, lipid nanoparticles

## Abstract

With the deepening of our understanding of adaptive immunity at the cellular and molecular level, targeting antigens directly to immune cells has proven to be a successful strategy to develop innovative and potent vaccines. Indeed, it offers the potential to increase vaccine potency and/or modulate immune response quality while reducing off-target effects. With mRNA-vaccines establishing themselves as a versatile technology for future applications, in the last years several approaches have been explored to target nanoparticles-enabled mRNA-delivery systems to immune cells, with a focus on dendritic cells. Dendritic cells (DCs) are the most potent antigen presenting cells and key mediators of B- and T-cell immunity, and therefore considered as an ideal target for cell-specific antigen delivery. Indeed, improved potency of DC-targeted vaccines has been proved *in vitro* and *in vivo*. This review discusses the potential specific targets for immune system-directed mRNA delivery, as well as the different targeting ligand classes and delivery systems used for this purpose.

## 1 Immune cell targeting, why?

Vaccines represent a potent strategy in the prevention of infectious diseases and play a significant role in reducing or eradicating the prevalence of severe illnesses globally (1, 2). Currently most vaccines primarily consist of pathogen-derived antigens, including purified proteins or inactivated pathogens. To enhance their efficacy, they are often formulated with adjuvants, which play a critical role in stimulating innate immunity immediately after immunization (3). These vaccines also induce the development of antigen-specific memory B and T cells, providing long-lasting protection to vaccinated individuals (4). It is estimated that traditional vaccines (e.g., live-attenuated, inactivated, and subunit types) save approximately 3 million lives every year (2). Nonetheless, vaccine development against emerging and re-emerging diseases is particularly challenging, as for Antimicrobial Resistant (AMR) bacteria and challenging-to-treat viruses (2). Moreover, the development and deployment of conventional vaccines are considerably long - it takes 5 to 10 years to develop a vaccine for an infectious agent. Therefore, scientists continue to explore new vaccine platforms to shorten the development cycle. It is however crucial that those platforms maintain the ability to also induce cell-mediated immune response to generate a strong and long-lasting protective immunity against pathogens.

Recently, mRNA-based vaccines have transformed the vaccine landscape, providing attractive breakthroughs in vaccine design and efficacy. Indeed, mRNA-based vaccine strategies stand out for several reasons linked to their usage. The first regards safety, as there is no potential risk of infection or insertional mutagenesis due to mRNA’s non-infectious and non-integrating nature (5, 6). In addition, *in vivo* delivery can be achieved through nanocarriers (for instance, lipid nanoparticles, LNPs) that enable RNA protection from degradation, fast uptake and cytoplasmic delivery, while anti-vector immunity is avoided (7). More, mRNA vaccines have the potential for rapid, cost-effective and scalable manufacturing mainly due to the high yields of *in vitro* transcription reactions. Furthermore, mRNA sequence can be quickly modified for personalized treatments or to urgently adapt to emerging pathogens e.g. during a pandemic or epidemic outbreak (5, 6). Engineering of the mRNA molecule and of effective mRNA delivery systems has enabled this technology to grow exponentially, and the platform still offers room for improvement, as it can be easily modified to modulate its half-life as well as its immunogenicity profile (8, 9). Indeed, some immunological elements still need to be improved to enhance mRNA vaccination outcome. For example, in the context of SARS-CoV-2, the efficacy of mRNA vaccines was demonstrated to be > 90% (10, 11), but the durability of the antibody response against spike protein seems to be considerably short and requires the administration of sequential booster doses to maintain protection against the infection. It is predicted that individuals lose more than 99% of humoral immunity relative to peak immunity after 8 months of the second dose of both BNT162b2 (Pfizer-BioNTech) and mRNA-1273 (Moderna) (12). Another aspect that is highly relevant for patient compliance is the emergence of side effects, such as injection site pain, fever, fatigue, headache and diarrhea following mRNA vaccination (13).

**Figure 1:**
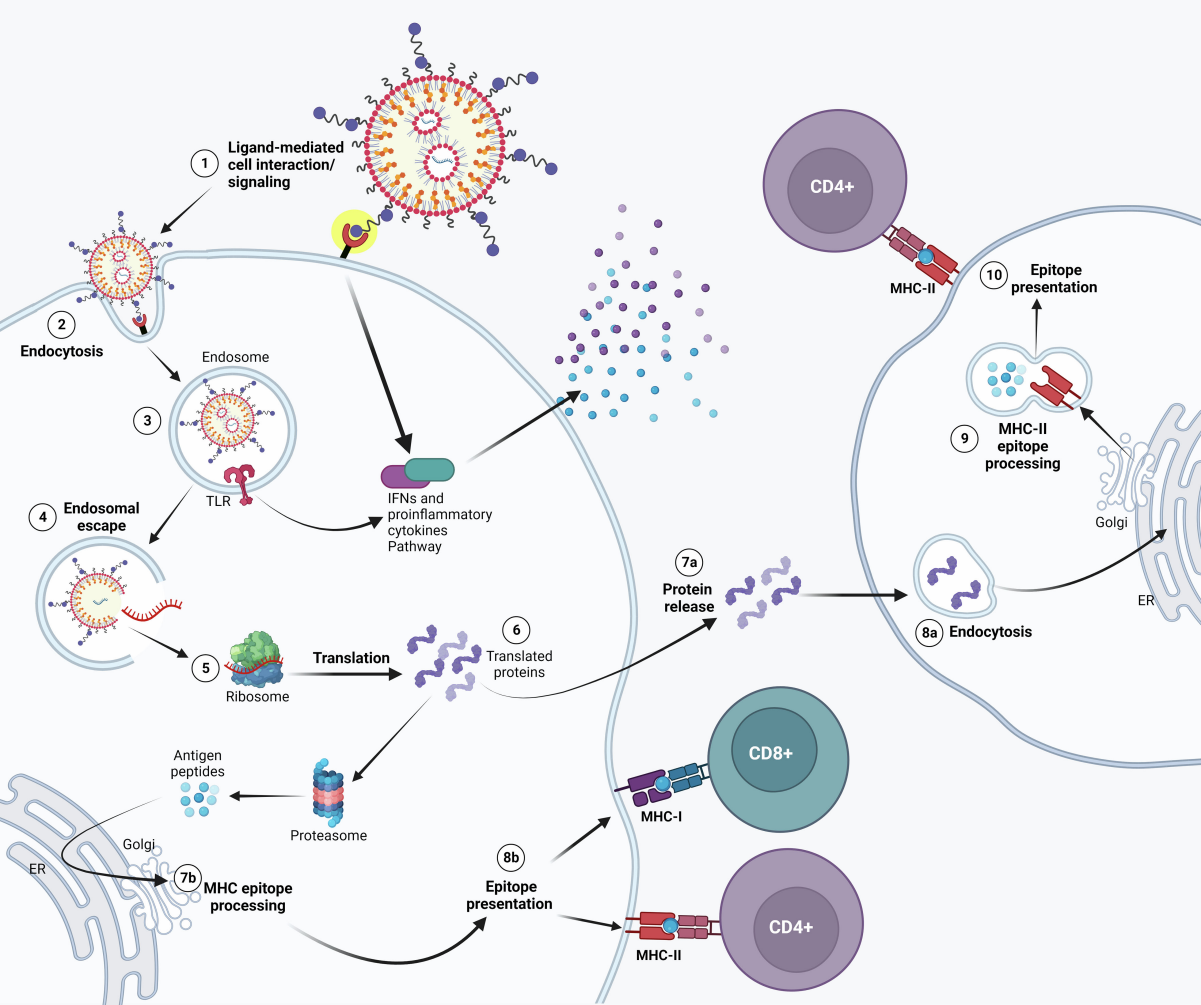
Possible mechanism of action of APC-targeted LNP-mRNA. Targeted mRNA-LNP-cell interaction is mediated by specific ligands that bind to APC cell surface receptor. Receptor activation might lead to IFNs and other cytokine/chemokine production (1). Following endocytosis (2), mRNA in the endosome can interact with membrane-bound Toll-like receptors (3). Triggering of TLR activates signal transduction pathways that selectively lead to production of Type I IFNs and/or pro-inflammatory cytokines. (4) Entrapped mRNA then undergoes endosomal escape and is released into the cytosol where it is translated by ribosomes (5). Upon translation, the protein can be secreted out of the host cell or processed into smaller peptides by proteasome. Proteins secreted extracellularly (7a) can be taken up by other antigen presenting cells (APCs) (8a) and then degraded into peptides subsequently presented on the cell surface by MHC class II molecules (9) for recognition by CD4^+^ T lymphocytes (10). Alternatively, translated proteins are degraded by the proteasome into peptides in the same cell (6). The generated antigenic peptides are then transported into the endoplasmic reticulum and loaded onto major histocompatibility complex (MHC) class I and/or Class II molecules, as less common pathway (7b). The loaded MHC - peptide epitope complexes are then presented on the surface of the APC and may bind the T cell receptor (TCR) of CD8^+^ and/or CD4^+^ T lymphocytes (8b).

To overcome these issues, mRNA targeted delivery comes up as a promising improvement. Briefly, the purpose of an mRNA vaccine is to make cells process delivered mRNA and release the translated antigen or present the epitopes on the surface of the cells (**Figure 1**). Indeed, after intramuscular administration, a transient local inflammation drives the recruitment of immune cells that can uptake the mRNA-LNPs and migrate to local lymph nodes where T cell priming occurs (14). In particular, administration of mRNA-LNPs induce rapid and local infiltration of neutrophils, monocytes, and dendritic cells (DCs) to the site of administration and the draining lymph nodes (LNs) (15). While these cells efficiently internalize mRNA-LNPs, mainly monocytes and DCs translate the mRNA and upregulate key co-stimulatory receptors (CD80 and CD86), and cytokine production (16). However, the fate of mRNA-LNPs is not restricted to immune cells. Biodistribution studies show that most of them are taken up by local muscle cells, while a residual part reaches the blood stream to end up mainly in the liver and spleen, contributing to the emergence of off-target effects (17). In this sense, the selective targeting of mRNA into immune cells could enhance the efficacy of the immune response while reducing the off-target biodistribution. Recently, evidence arose that migration to secondary lymphoid organs was the main driver for immune response compared to mRNA expression in the muscle (18). As a result, targeting mRNA delivery to immune cells and eventually to the lymph nodes could increase the magnitude and lifespan of protection and eventually allow a reduction in the required mRNA-LNP dose, minimizing side effects.

In this context, antigen presenting cells (APCs), in particular dendritic cells (DCs), stand out as the most promising candidates for targeted mRNA delivery due to their unique ability to initiate adaptive immunity by priming of naïve T cells (19). Both prophylactic and therapeutic vaccines require robust CD4^+^ and CD8^+^ T cell responses and the concept of directed DC targeting enhances antigen presentation to these specific cells. Moreover, it has been shown that DC targeting also enhances and accelerates specific antibody responses even at low antigen doses (20).

DCs fulfill the three essential signals required for T cell priming and differentiation. Signal 1 involves the presentation of antigenic peptides with MHC class I and class II molecules, which enables them to prime both CD4^+^ and CD8^+^ T cell responses. Signal 2 is provided by the upregulation of costimulatory molecules, such as CD80 and CD86, on activated DCs, which interact with CD28 on naïve T cells (21). Signal 3 encompasses the secretion of cytokines like IL-12 and type I IFNs, as well as the expression of surface molecules like CD70 and OX40L that regulate the proliferation, differentiation, and survival of primed T cells (22, 23). In addition, a key feature for DCs to exert their immunological function (either of immunity or tolerance) is their ability to migrate from the site of antigen uptake to the sites where immune responses are initiated, such as the T-cell zones of lymph nodes (24, 25).

Targeting can be achieved using ligands that specifically interact with cellular receptors on the surface of specific cells (26). The engagement of different receptors influences the type of induced immune response and can be exploited to obtain a specific desired outcome. In this frame, DCs exhibit plasticity and can be manipulated to elicit specific immune responses since they express several receptors that could be targeted for vaccine delivery (27). These receptors include pathogen recognition receptors (PRR) like C-type lectin receptors (CLRs), a superfamily of proteins that interact with several types of sugars on the surface of pathogens and have a crucial role in the capture and presentation of the antigen, and Toll-like receptors (from TLR1 to TLR10), both primarily involved in the recognition of pathogen-associated molecular patterns (PAMPs) (28). DCs also express Scavenger receptors like CD36 (29, 30) involved in the uptake of a variety of ligands, including lipids, apoptotic cells, and microbial components as well as several other receptors such as chemokines receptors, complement receptors and Fc receptors, all regulating antigen presentation functions (31). In the sections that follow, we provide a more detailed description of such receptors and their respective ligands.

Overall, the targeting of such receptors on DCs can enhance antigen uptake, presentation, and immune response activation. This opens the possibility to develop vaccines that can elicit stronger humoral (antibody-mediated) responses, cellular immune responses, or a combination of both, according to the pathogen’s nature and the desired immunological outcome. Indeed, this strategy has already been applied successfully to subunit vaccines (32–34). Altogether, DC targeting could potentially not only improve mRNA vaccine efficacy, but also expand the range of pathogens for which they do effectively work, including those that have developed mechanisms to bypass the immune system, which are some of the most challenging to address (35).

It is also noteworthy that not only DC subsets, but also other cell types involved in the immune response, such as macrophages, could be interesting targets for this approach. These immune subpopulations may express some common receptors, along with specific receptors unique to each. In the following paragraphs we review more in detail target cells and their receptors of interest, as well as formulation strategies to achieve selective mRNA targeting.

## 2 Target cells

DCs can be classified into several sub-populations based on their distinct functionalities and expressed receptors. These sub-populations have important roles in triggering and modulating immune responses. The complexity and versatility of the human DCs’ phenotype and functionality pose challenges in their precise classification, which is continuously being revised (35). The main subsets of dendritic cells are conventional DCs (cDC1 and cDC2), plasmacytoid DCs (pDC) and follicular DCs (FDCs).

### 2.1 Conventional dendritic cells (cDCs)

cDCs originate in the bone marrow from hematopoietic stem cells that give rise to intermediate progenitors called common DC progenitors – restricted to the DC lineage. The differentiation of common DC progenitors into pre-cDCs depends on the binding of the growth factor FMS-like tyrosine kinase 3 (Flt3) to its ligand. Pre-cDCs leave the bone marrow, travel through the bloodstream, and undergo maturation into cDCs within lymphoid and peripheral organs such as the spleen, lymph nodes, intestines, and lungs (36). Differentiated cDCs in both lymphoid and peripheral organs highly express MHC molecules for antigen presentation and CD11c. cDCs can be further classified into two subgroups: cDC1 and cDC2. These subgroups express different markers and rely on distinct transcriptional factors for their development (37).

#### 2.1.1 Conventional dendritic cells 1 (cDC1)

cDC1 cells are found in peripheral tissues as well as lymphoid organs. Mouse and human cDC1s express the chemokine receptor XCR1 (X-C Motif Chemokine Receptor 1) and the CLRs DNGR-1. Moreover, cDC1s in lymphoid organs express CD8α, while those in peripheral tissues express CD103 (38). The differentiation of cDC1s is regulated by transcription factors such as Irf8, Batf3, Id2, and Nfil3 (39). cDC1s are specialized in efficiently presenting exogenous antigens to CD8^+^ T cells, thereby promoting cytotoxic T cell immunity. This process, known as cross-presentation, requires MHC-I molecules for antigen loading (21, 40). Studies in mouse models have demonstrated that cDC1 play a role in the early priming of CD4^+^ T cells in the context of tumor-derived antigens (41). This cell subset produces IL-12 to control microbial infections, are specialized in producing type III IFN (42) and promote T helper (Th) 1 cell differentiation (38). However, studies *ex vivo* have demonstrated how cDC1s from blood, lymphoid organs, and lungs stimulate the polarization of naïve CD4^+^ T cells into both Th1 and Th2 cells (43).

#### 2.1.2 Conventional dendritic cells 2 (cDC2)

Mouse and human cDC2 cells are present in peripheral tissues and lymphoid organs, particularly concentrated at the boundary of T-B cell zones (44). The majority of cDC2s express a high level of CD11b. Additionally, cDC2s that are present in lymphoid organs like lymph nodes and spleen express CD4 (45), while those in peripheral organs such as the lung and intestine express CD24 and CD103 respectively (46). The differentiation of cDC2s relies on the presence of IRF4, Klf4, Zeb2, IRF2, RelB, Ikaros, or Notch and it results in the preferential activation of CD4^+^ helper T cell (Th) responses (47). Recently, single-cell RNA sequencing on murine DCs has identified two additional subsets of cDC2s: Tbx21 (T-BET)^+^ cDC2a and Rorc (RORγT)^+^ cDC2b. Moreover, these findings were extended to human splenic DCs by demonstrating a parallel sub-division among human CD1c^+^ cDC2s (48). cDC2a and cDC2b have a high level of functional specialization. Indeed, cDC2a cells have a regulatory anti-inflammatory function, while cDC2b cells possess proinflammatory potential (49).

Additionally, a recent investigation proposed another distinction between cDC2 subpopulations. This heterogeneity is characterized by the presence of two distinct subpopulations in human, now known as DC2 and DC3 (50). Single-cell RNA-seq analysis has revealed that both subpopulations exhibit similar gene expression patterns related to inflammasome signaling under steady-state conditions (51). However, they differ significantly in their production of IL-1β in response to TLR stimulation (52). DC2 subpopulation releases lower levels of inflammasome-dependent IL-1β but has a stronger capacity to elicit CD4^+^ T cell responses compared to DC3. The T cell responses induced by DC2 are predominantly skewed toward a Th1/Th17 phenotype. Thus, it appears that DC2 subpopulation has the ability to enter a state of hyperactivation, resulting in enhanced T cell stimulatory capabilities (50). Last, this distinction between DC2 and DC3 in human has also been conversely observed in mice (53).

The major role played by the cDC2s, both in human and mice, consist in the recognition and presentation of foreign antigens to CD4^+^ T cells, supporting their differentiation into Th2 and Th17 cells, and facilitating T helper cell-mediated immune responses (54). The differentiation of CD4^+^ T cells into Th17 relies mainly on the ability of blood and lung cDC2s to secrete IL-23 (55, 56). Moreover, it has been shown that, like cDC1s, human cDC2s in the blood, lymphoid organs, skin, and lung have the ability to induce the polarization of naïve CD4^+^ T cells into Th1 and Th2 cells *ex vivo* (57). Moreover, human cDC2s in the blood, lymphoid organs, lung, and skin are characterized by their elevated expression of activin A and OX40-ligand and are considered highly effective inducers of T-follicular helper (Tfh) cells (58–60). In terms of antigen presentation, cDC2s in the blood, lymphoid organs, lung, and skin are equally efficient as cDC1s in cross-presenting soluble protein antigens *ex vivo* (61). They also play a role in stimulating the differentiation of cytotoxic CD8^+^ T cells (62).

### 2.2 Plasmacytoid dendritic cells (pDCs)

pDCs are a small subset of DCs that are also essential players in the field of vaccination, contributing to immune cell activation and polarization, in the context of antiviral immunity. Human pDCs naturally overexpress high levels of endosomal nucleic acid-sensitive Toll-like receptors (TLRs), such as TLR7 and TLR9, which detect single-stranded RNA and unmethylated DNA carrying CpG motifs, respectively (63). pDCs answer to nucleic acids with extensive secretion of type I IFN, involving IFN-β and different subsets of IFN-α. Moreover, pDCs also secrete type III interferons (IFN-λ) and additional cytokines (e.g., TNF-α) and chemokines (64–66). Further investigations and clinical studies are needed to comprehend the capabilities of pDC targeted vaccines and their implementation in fighting infectious diseases and cancer. It is worth mentioning that pDCs are exceptional producers of type I IFN, which play a crucial role in initiating antiviral immune responses. By targeting pDCs, vaccines can prime rapid and robust production of these interferons, enhancing the organism’s ability to fight viral infections early. Therefore, the specific role of pDCs and their adjuvant capacity and immune modulation abilities could provide a valuable reason for optimizing pDC- targeted vaccine formulations in the future. Nevertheless, conventional DCs are generally considered more efficient in antigen presentation and T cell activation as compared to pDCs and for that reason they remain the principal target for vaccine delivery.

### 2.3 Follicular dendritic cells (FDCs)

FDCs are a specialized subset of DCs that have a crucial role in the immunological response, especially in lymph nodes and other lymphoid tissues and are considered pivotal in generating a strong and long-lasting immune response (67). Their major function is to facilitate B-cell activation through the presentation of antigen-antibody immune complexes, following clonal expansion of specific B-cell clones. Another key role of FDCs is the establishment of specialized microenvironments in lymph nodes known as germinal centers, where B cells experience somatic hypermutation and affinity maturation (68).

It is worth noting that FDCs present some differences from cDCs not only at the functional but also at the cellular lineage level. Indeed, FDCs origin is distinct from cDCs, as they do not arise from hematopoietic cells but from mesenchymal lineage. FDCs are not conventionally recognized as antigen-presenting cells. They express unique surface markers like CR2/CD21 and CR1/CD35 (complement receptors). CR1-associated proteins are involved in signaling and contribute to their unique functions within the germinal centers. Another difference is that FDCs do not express some of cDCs markers such as MHC class-II. On the other hand, they also express CLRs (e.g., CD209 and CD206) that facilitate the uptake of glycosylated antigens (69).

Targeting FDCs appears as a valuable strategy due to the expression of distinctive markers that make it accessible for specific, focused immunomodulation, as well as the opportunity for a synergistic targeting by leveraging shared markers. Within this framework, targeting specifically FDCs could lead particularly to a great optimization of humoral response to induce a more potent and targeted antibody response. Indeed, as FDC contribute to the generation of plasma cells and B cell memory, this approach could be valuable for pathogens that primarily elicit immune response through antibodies.

### 2.4 Langerhans cells (LCs)

Langerhans cells (LCs) are classified as member of DC cells/macrophages family. In particular, LCs were first considered a subset of DCs due to their capacity to migrate to skin-draining lymph nodes (70). However, recent studies in mice have shown that LCs are a subset of tissue-resident macrophages that acquire a DC-like phenotype and function upon further differentiation in the skin (71, 72). These cells are localized in the epidermidis, where they reside in close association with keratinocytes and form a complex network that is intended to induce the first reactions against pathogens encountered in the skin (73). Indeed, human and mice LCs are specialized at sensing the environment and at sampling the outermost layers of the skin (stratum corneum) thanks to the ability to extend their dendritic processor through tight junctions (74). These peculiar features suggest the strategic importance of LCs as immune sentinels and as a first line of defense. Indeed, following antigen recognition and processing, LCs elicit an immune response in the skin-draining lymph nodes where they promote efficient T cell response, humoral immunity by B cell activation and expansion of Tfh cells (75–78). Moreover, the skin is considered a promising vaccination access point for the administration of targeted delivery vaccines due to the high density of LCs. Indeed the skin displays higher immune cell density compared to the muscle or the subcutaneous layer, what offers several advantages: 1) lower vaccine doses; 2) minimally invasive administration and 3) increase in patient compliance (79).

Mouse and human LCs express an exclusive surface marker to consider as target for delivery: the Langerin receptor (CD207). Langerin is a C-type lectin that recognizes mannose, *N*-acetyl-glucosamine and fucose structures on viruses, bacteria and fungi as well as self-antigens (80, 81). Following uptake, antigens are transported into the endosomal compartment of LC cells where the acidic environment causes a release of the antigen and the subsequent recycle of the receptor on the cell surface (82). Altogether, these findings underly the potential of targeting LCs in skin immunization.

### 2.5 Macrophages

In addition to DCs, macrophages play a critical part in modeling the adaptive immune response (83–85). Their primary role is to phagocyte and destroy foreign substances, such as pathogens or dead cells. After the early immune response, macrophages play a role in tissue remodeling and repair. They clear away debris and promote healing by delivering growth factors and extracellular matrix components. This process is essential for rebuilding tissue integrity and function. Nevertheless, their functions go beyond this initial response and impact the development of the adaptive immune response, since they can also present antigens to T cells and secrete cytokines to tune immune responses.

Macrophages express several receptors that that could be targeted for vaccine delivery such as TLRs (28), mannose receptors (86), scavenger receptors (29) and Fc receptors (31). Targeting this cell population is particularly interesting for respiratory infections, since alveolar macrophages are the first cells to present pathogens to pulmonary draining lymph nodes (87). In fact, mannosylated nanoparticles present higher accumulation on alveolar macrophages through the interaction with mannose receptor (MR) and result in upregulation of costimulatory molecules and enhanced production of proinflammatory cytokines IL-1β and TNF-α (88). However, it is worth pointing out that in general DCs have a stronger ability to elicit antigen-specific immune responses compared to macrophages as they have superior antigen presentation ability, whereas macrophages act primarily as phagocytes. Moreover, differently from DCs, most macrophage populations present very restricted ability to migrate from peripheral tissues to lymph nodes. This migration is critical for effective immune activation and the establishment of adaptive immune responses. Therefore, macrophages are generally considered less favorable than DCs for targeted delivery. Yet, it is worth noting that macrophages can interact with different immune cells, including T cells, B cells, and natural killer (NK) cells. In this sense, targeting macrophages could facilitate the crosstalk between these cell types, which in turn, could result in a more integrated and comprehensive immune response. Moreover, macrophages reside in several tissues and organs throughout the body and by specifically directing the vaccine to engage with tissue-resident macrophages, it could be possible to initiate localized immune responses in specific regions. This approach could be particularly advantageous for infections that predominantly affect specific tissues or organs (83, 84, 89).

Overall, it is important to note that every APC has both benefits and limitations, and the ultimate choice of which APC to target would depend also on the objectives of the specific vaccine and the features of the pathogen or antigen to be targeted. In addition, a combination of multiple APCs, such as DCs and macrophages, could be used to elicit synergistic effects and generate a more robust and versatile immune response.

## 3 Target receptors

The type and extent of the immune response depend on the context in which the antigen is captured, which includes which receptor is used for internalization. Therefore, the success of a targeted delivery platform depends on choosing the right pair of ligand-receptor. The receptor should be selected according to the expression pattern of the target cell, its endocytic capacity and its ability to facilitate intracellular antigen delivery to compartments specialized in processing and loading on MHC (90). Each receptor determines the antigen intracellular pathway and, more specifically, even the binding site on the receptor can influence which route will get activated.

### 3.1 C-Type lectins as targets for targeted mRNA-delivery

#### 3.1.1 Generalities on C-type lectins

CLRs are a superfamily of more than 1,000 proteins classified into 17 subgroups (I–XVII), according to their homology and phylogeny (91). CLRs are proteins primarily expressed on myeloid cells where they perform various roles but effectively function as recognition receptors for self and non-self antigens (91–93). CLRs are abundantly expressed on the surface of various immune cells, including DCs, macrophages, and neutrophils. They are involved in recognizing pathogens, initiating immune responses, and modulating immune cell interactions. Predominantly transmembrane proteins (93), they act as PRR recognizing PAMPs, but also damage-associated molecular patterns (DAMPs) (92, 94, 95). From a structural point of view, CLRs possess a conserved structural motif arranged as two protein loops stabilized by two disulfide bridges at the base of each loop (91). Those domains all have similar folds and are named Carbohydrate Recognition Domain (CRD). They canonically recognize carbohydrates through interactions with conserved Calcium-chelating binding sites, although they may also interact with non-sugar ligands (96–99).

CLRs are key actors of viral, bacterial, and fungal immunity, as they are involved in the recognition of carbohydrate-containing structures associated with these pathogens, thereby facilitating their identification and phagocytosis (100). After binding of pathogens by CLRs, the intracellular routing of antigens has various outcomes depending on the CLR and the immune cell (101). For example, while MR-mediated endocytosis leads the antigen to lysosomes, MGL-1 (macrophage galactose-type C-type lectin 1, Clec10a)-facilitated internalization in murine Bone Marrow-derived Dendrtici Cells (BMDCs) drives antigens to late endosomes (102, 103). Once PAMPs are presented in sufficient amount, multiple CLRs initiate signaling responses. CLRs signaling is a complex process that can itself interfere with other PRR signaling pathways. Broadly speaking, CLRs signaling can be divided in two groups: the first group transduces intracellular signals through the integral immunoreceptor tyrosine-based activation (ITAM)-like motif (Clec-2, Dectin-1), or via association with ITAM-bearing FcRγ adaptor molecules (Dectin-2, Mincle, BDCA-2) (104). Upon phosphorylation, the ITAM motif recruits and activates Syk, what induces transcription of pro-inflammatory cytokines by activating subunits of the transcription factor NF-κB complex (105). Details of CLR signaling and their immunological outcomes have been brilliantly reviewed elsewhere (100, 105). Interestingly, although many CLRs share this signaling pathway, they all lead to specific responses; recently, fascinating reports were published revealing how kinetics and information transfer of similar lectins allow for their different biochemical outcomes (31, 106). The second group of CLRs, on the other hand, bear on their cytoplasmic end an Immunoreceptor Tyrosine-based inhibition (ITIM) motif (e.g. MICL) (100). This review focuses on the first group of signaling CLRs.

In addition to signaling, CLR-ligand interactions on the surface of immune cells can result in improved phagocytosis (92, 107). Interestingly, some CLRs like Dectin-2 (Clec6a) are shown to improve phagocytosis although they do not directly participate in the phenomenon, but likely trigger signaling which in turn catalyzes the engulfment process (108). In summary, CLRs are considered attractive targets for targeted antigen and mRNA-delivery (34) due to their broad expression on antigen presenting cells, their selectivity and their role in internalization, antigen processing and immune activation.

#### 3.1.2 The mannose receptor as a case-study for targeting strategies

A CLR that has received much attention for targeting purposes is the MR (CD206, MR). CD206 is an endocytic receptor expressed on macrophages and DCs. It favors the cross-presentation of soluble ligands (109, 110) that display mannosides (from simple mannose to higher mannan structures) as well as fucose and sulfated LacdiNAc (109, 111). Since MR can interact even with simple monomeric mannosides, several strategies have been put in place to incorporate MR-targeting ligands and render mannose-based targeting one of the most common strategies for CLR-targeting. As early as in 2006, White et al. used mannosylated liposomes to increase OVA uptake by monocyte-derived DCs (moDCs) *in vitro* model (112). Likewise, mannosylated liposomes showed 4-fold higher eGFP expression levels in splenic DCs than non-mannosylated controls in mice (113). The authors confirmed the receptor-mediated nature of the internalization by injecting LucDNA mannosylated liposomes prior to eGFP mRNA mannosylated liposomes and seeing that the eGFP expression was almost completely reduced (113). Indeed, a myriad of reports show that mannose-based strategies increase internalization (114) and transfection (113) of mRNA vaccines in immune cells through a receptor-mediated mechanism. Last, a first clinical trial with a MR-targeted cancer vaccine was reported in 2011 (115), but so far we are not aware of any clinical activities for MR-targeted mRNA vaccines.

**Figure 2:**
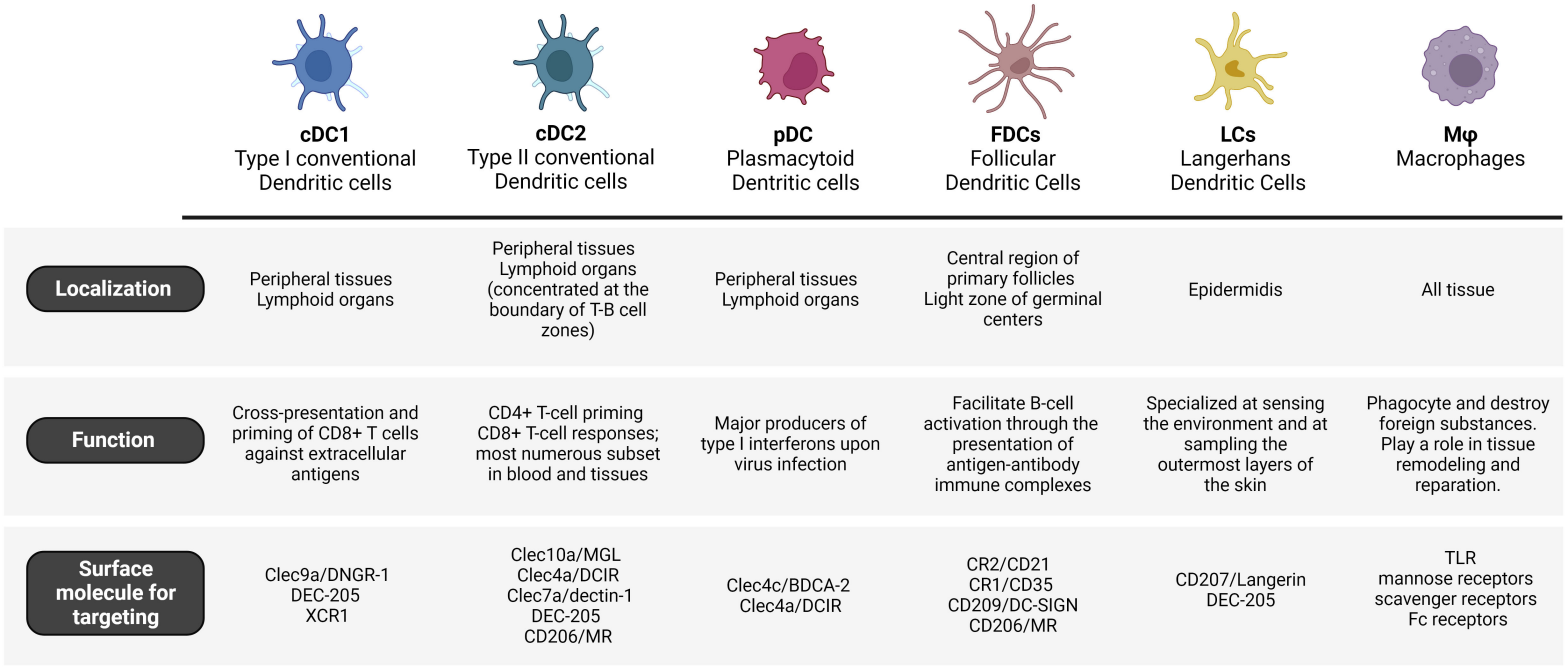
Overview on APC cells and surface receptors for targeting approaches.

Though, it is worth noting that most studies confirm the receptor-mediated nature of the internalization process through competitive binding assays using mannan and, therefore, cannot individualize which exactly receptor is being used. Although the strategies described in the previous paragraph may result in targeting CD206, there are other receptors that can recognize mannosylated ligands (116). The simplicity of the ligands used, and the variety of C-type Lectins and their recognition patterns indicate that using simple oligomannosides will not make for a selective MR-targeting at subtype level. Many cell types display receptors that are able to recognize simple mannose (116) and this might render simple mannosylation an inefficient targeting strategy, and advocates for the development of more specific ligands, or the targeting of CLRs with restricted expression profile. For instance, vaccines that target MR may also bind DC-SIGN due to similar ligand binding profiles, as DC-SIGN is a CLR that also recognizes terminal mannosides (117). As detailed next, this receptor has been broadly considered for targeted antigen delivery (118, 119), but the synergy might not be cooperative, and the expression patterns differ (**Figure 2**), thus highlighting the need for specific ligands when designing targeting strategies.

#### 3.1.3 Other C-type lectins

DC-SIGN is a calcium-dependent lectin found on DCs and macrophages subpopulations in human (120, 121) that binds high mannosylated glycoconjugates and fucose-containing antigens (122). It plays a central role in the recognition of pathogens and mediates DC-T Cell interaction by facilitating T Cell adhesion to scan the DC surface for the presence of peptide antigens (123). It is believed that DC-SIGN induces intracellular signaling that modulates signaling of other PRRs such as TLRs (124). This feature could be particularly beneficial for vaccine strategies in terms of adjuvanticity. Indeed, DC-SIGN has been used as target for antigen delivery through glycan modified liposomes, which resulted in higher antigen accumulation on DCs and increased T Cell activation compared to non-mannosylated control (125, 126). It is worth noting that DC-SIGN ligand design is an active field of research, both with glycomimetics and drug-like small molecules (119, 127, 128).

Another CLR attracting attention is DEC-205 (CD205). Expressed on a range of DC subsets, monocytes and LCs (129), but also on B cells, T cells and NK cells at lower levels (130), DEC-205 is involved in endocytosis and increases MHC II presentation (131). Although its immunological functions have been investigated already in 1995 (129), DEC-205’s structure has been solved only recently (132), and its set of ligands still hasn’t been fully resolved. CD205 has been shown to recognize apoptotic and necrotic signals (133), although the molecular mechanism and selectivity with which it recognizes its ligands remain poorly elucidated (134). Indeed, most of DC205-targeting attempts rely on the use of antibodies or antibody-fragments, yielding efficient DC-targeting and enhanced antigen cross-presentation (135, 136). Although these examples consist in antibody-antigen fusion proteins, two examples of relevance for this review are: an anti-DEC-205-targeted PLGA particle showed enhanced cross presentation of a melanoma-associated antigen (137) and a DNA vaccine encoding for an anti-DEC-205 Ab fused to the antigen showed improved response compared to non-targeted antigen (135). Of note, in 2011 a clinical trial was conducted for an anti-HIV DEC-205-targeted vaccine (138).

Dectin-1 is a CLR that identifies ligands independently of Ca^2+^ and promotes particle uptake through actin-dependent phagocytosis (139). It specifically recognizes soluble and particulate β(1-3) and/or β(1-6)-linked glucans with different affinities that depend on the degree of side chain branching and polymer chain length (140). Dectin-1 is involved in cellular activation through an ITAM-like motif in its cytoplasmic tail. In this sense, its optimal activity is reached in collaboration with other PRRs (141). This receptor is thought to be a costimulatory molecule as it can bind to both CD4^+^ and CD8^+^ T cells and increase their proliferation (142). It is expressed by DCs, macrophages, monocytes, neutrophils and a subset of T cells, and very highly expressed on portals of entry like intestine and lungs (139). The conjugation of a small interfering RNA (siRNA) with β-glucan polysaccharide resulted in Dectin-1-mediated accumulation in Dectin-1-positive cells in PBMCs (143).

As described previously, Langerin (CD207) is an attractive target for receptor-mediated antigen delivery as it is highly expressed by LCs (144), dermal DCs (145, 146) and other DC subtypes (147). Several attempts have successfully resulted in LC targeting *in vitro* using antibodies (148) or small sugars (149, 150) as targeting ligands. Interestingly, Wamhoff et al. reported the discovery of a glycomimetic Langerin ligand and demonstrated its selectivity for Langerin^+^ cells using functionalized liposomes (149). Schulze et al. used this small Langerin ligand to selectively target nanoparticles (liposomes) to human LCs *in vitro* (151), and Rentzsch et al. proteins to LCs ex vivo (152). Such ligands can be used to functionalize nanoparticles which encapsulate drugs, antigens or toxins to be delivered into LCs (151).

Two other examples of CLR attracting attention for cell-specific targeted delivery are DNGR-1 (Clec9a) and MGL (Clec10a) (33) (**Figure 2**). DNGR-1/Clec9a was shown to be selectively expressed on cDC1 in humans (153). Clec9a is an appealing target as it has been shown that specific interaction with this receptor promoted humoral immunity in non-human primate (154). Although, to the best of our knowledge, DNGR-1 targeting has never been applied to mRNA-vaccines, a recent example showed that a DNGR-1-specific peptide could target nanoparticles to Clec9a^+^ DC (155, 156). MGL, on the other was shown to be specifically expressed on cDC2 (CD1c^+^ DCs) in humans (157). It binds selectively to terminal GalNAc (158) and mucin-1 peptides with various glycosylation profiles (159). Heger et al. showed that using a Clec10a-binding glycopeptide, it was possible to selectively targeted CD1c^+^ cells (cDC2s) in PBMCs (157). Last, the authors did not observe that ligands for Clec10a alone induced activation or cytokine secretion by CD1c^+^ DCs (although it did lead to targeting), what is an interesting feature for the development of targeted immunotherapeutics.

Last, hDCIR (Clec4a) is a broadly expressed CLR, being found on all CD14^+^ monocytes, CD15^+^ granulocytes, all DC subsets (including pDCs) and B cells in peripheral blood, but not T Cells (104, 160–162). It is, to the best of our knowledge, not very clear what Clec4a binds to, but it is involved in HIV glycoprotein recognition (163). Targeting of antigens to DCIR using antibodies resulted in increased cross-presentation by LCs, blood mDCs and pDCs, and enhanced CD8^+^ T cell priming in human cells *in vitro* (164). The fact that Clec4a has a broader cell expression pattern makes it interesting, and its targeting could be used for different strategies as compared to the receptors with restricted expression pattern that we have mentioned before.

In conclusion, CLRs are attractive and versatile targets for targeted antigen delivery. With increasing knowledge in their structural and molecular biology, an ever-growing range of ligands and targets are becoming available. Of particular interest are those CLRs for which we can find selective ligands. When selective ligands are lacking, many examples rely on the use of antibodies although their generally high affinities may prove deleterious for endosomal escape (165). Last, the targeting of CLRs with restricted expression profiles (Langerin, XCR-1, Clec9a, Clec10a) allows for specific targeting to defined cell subtypes.

### 3.2 Other receptors for targeted mRNA-delivery

Although CLRs represent most of the targets presently described in the literature, there are other receptors that hold potential for targeted mRNA delivery to immune cells. Toll-like receptors (TLRs) play crucial roles in the innate immune system by recognizing PAMPs. TLRs are proteins expressed in particular by innate immune system cells, such as macrophages and DCs but they can also be expressed by some subsets of adaptive immune cells (28). TLRs signaling recruits specific adaptor molecules, which leads to the activation of the transcription factors NF-κB and IRFs, thus dictating the response’s outcome (166, 167). Many commercial adjuvants contain TLR-agonists (168). Recently, a nanoparticle was targeted to DCs using Pam3CSK4 (a TLR-2 agonist) as targeting ligand (169). The use of TLR agonists as targeting ligand represents an efficient way to target DCs, but the activation of these receptors also poses the risk of modulating the immune response to potentially increase inflammation. If harnessed, this could also improve the response to targeted vaccines.

X-C Motif Chemokine Receptor 1 (XCR1) is a chemokine receptor known to recognize XCR1 ligand (XCL1). It is selectively expressed on cDC1 (170). Being a specific ligand, vaccines using XCL1 as targeting ligands fused to antigens (rather than monoclonal antibodies) have been developed and showed improved efficacy (171, 172). An important communication from Fossum et al. compared Clec9a, DEC-205 and XCR-1 as targets in a single study, using DNA vaccines encoding for single chain variable fragments (scVf) fused to an antigen and injecting them into mice (173). Interestingly, they found that targeting to XCR-1 resulted in augmented IFN-γ^+^CD8^+^ T cell responses in both spleen and lung and stronger cytotoxicity, while targeting to Clec9A induced antibody responses with higher avidity and more neutralizing effect compared to XCR1 and DEC-205. This shows that although Clec9a and XCR1 are both cDC1 specific, targeting them results in different outcomes, thus showing that not only the cell subtype but also the receptor itself dictates the targeting’s outcome and efficacy.

Other immune cells have also been targeted for mRNA delivery. To deliver interleukin 10-encoding mRNA specifically to Ly6c^+^ leukocytes *in vivo*, Veiga et al. used mRNA-LNPs modified with anti-Ly6c (174). The authors saw a 20-fold increase of antigen expression in the target organ and a 10-fold decrease in the liver compared to the unmodified LNP. Likewise, CD4^+^ T-Cells that were targeted by mRNA-LNPs decorated with anti-CD4 antibody resulted in a 33-fold increase in antigen expression compared to the non-targeted mRNA-LNP (175).

In conclusion, potential targets for DC-targeted delivery are numerous, and offer the possibility to target DC subsets and to direct the LNP’s fate toward different routes and with different outcomes (**Table 1**). Targeting receptors expressed on multiple subsets may also magnify the desired immune response by multiplying the number of targeted cells. Last, it is worth noting that although most targeting strategies target a specific receptor, designating a specific target is not strictly necessary. Indeed, Jung et al. reported the discovery of peptide-targeted chitosan particles, where the targeting peptide was chosen for its ability to interact with BMDCs without knowing the peptides’ mode of action and binding partner (182). Targeting was confirmed *in vitro*, as uptake increased in DCs but not in control cells (myoblasts).

**Table 1:** Examples of immune cell targeting, their targeting strategy and outcome.

Target	Targeting Ligand	Model	Outcome	Ref
MR	anti-MR mAb	Human	Induced significant cellular and humoral response in Phase I clinical trial	(115)
MR	Oligo-mannosylated cholesterol	Mice	Improved *in vivo* humoral response	(176)
MR	Tri-mannose	Mice	Induced activation of splenic DCs and antitumor T cell response	(114)
DEC205	anti-DEC-205 mAb	Mature moDCs	Increased DC-uptake and increased presentation to CD8^+^ T Cells	(137)
Langerin	Glycomimetic Langerin ligand	Ex vivo human skin	Fast and specific LC-specific uptake	(132)
Clec9a	anti-Clec9a mAb	Mice, non-human primates	Increased humoral response, without adjuvant	(154)
Clec10a	Glycosylated peptide	hPMCs	Specific cDC2 uptake	(157)
Dectin-1	β-glucan	Mice	Induced potent Th1 and Th2-type responses	(177)
Dectin-2	Anti-Dectin-2 mAb	Mice	Increased CD8^+^ T Cell Response	(178)
DC-SIGN	anti-DC-SIGN mAb	Pigs	Production of antigen-specific CD4 T cell response, with Th1 polarization	(179)
DC-SIGN	Fucose-containing Lewis-type glycans	Human	100-fold increase presentation to CD4 and CD8 T cells compared to non-targeted control	(180)
DC-SIGN	Mannosides	moDCs	Induced Th1-type immune response	(181)
Clec4a	Anti-Clec4a mAb	hPBMCs	CD8^+^ T Cell cross-priming	(164)
XCR-1	DNA-encoded scFv-Ag fusion	Mice	augmented IFN-γ^+^CD8^+^ T cell responses	(173)
TLR-2	Pam3Cys	Mice	Reduced *Mycobacterium Tuberculosis* burden in the lung	(169)
Ly6c	Anti-Ly6c mAb	Mice	Targeted expression of IL-10 in Ly6c^+^ inflammatory leukocytes	(174)

## 4 Nanoparticles as targetable mRNA delivery vehicles

### 4.1 Vehicles and delivery systems

One of the main challenges in the development of mRNA vaccines is its intracellular delivery. The mRNA inevitably needs to arrive in the cytosol to meet ribosomes and finally get translated into the antigen. However, naked mRNA is not able to cross the plasma membrane and is susceptible to degradation by endonucleases, which are widespread in physiological fluids and tissues (183). For instance, the lifetime of naked DNA plasmid in blood is only a few minutes (184). To overcome this issue, mRNA can be associated to carriers that protect it from degradation and enable intracellular delivery.

To this end, many delivery systems have been used. They have been conveniently divided into viral (185) and non-viral vectors. The use of viruses takes advantage of their naturally-evolved ability to efficiently transfer genetic material into cells, which renders high translation efficiency (186). Nevertheless, viruses have their own tropism and not always meet the therapeutic needs. Naturally, they induce potent immune responses that can translate into reactogenicity and harm the therapeutic efficacy. More, this could have an impact on transfection efficiency in repeated immunization schedule. This may eliminate both vector and transfected cells, decreasing the intensity and duration of antigen expression (185). To overcome the antivector immunity-dependent decrease of response related to viral vectors, alternative technologies have been used, such as RNA-peptide conjugates (187, 188), polymers (189) and lipid nanoparticles (7). Nanoparticles, in particular lipid-based, have emerged as the currently preferred non-viral vector and represent a safer and more versatile alternative.

An ideal non-viral vector should efficiently bind to the mRNA to enable good encapsulation, protect it from enzymatic degradation, facilitate cellular uptake at the target cell and promote endosomal escape so the genetic material reaches the cytosol. In this sense, nanoparticles offer the possibility to tailor their properties to optimize their performance as transfection agents. Lipid nanoparticles (LNPs), for instance, have evolved from liposomes and lipoplexes to become more efficient mRNA vectors (190). The main advance in the LNP composition in relation to other lipid-based systems is the advent of ionizable lipids in their composition, as opposed to cationic lipids. Ionizable lipids are positively charged at acidic pH but neutral at physiological pH. This feature greatly improved the drawbacks inherent to permanently cationic lipids, like rapid elimination, poor tolerability and cellular toxicity associated to the positive charge (190). Ionizable lipids play a decisive role on the mRNA intracellular delivery by facilitating endosomal escape as well as on the mRNA encapsulation during manufacturing. Upon acidification in the endosomes, the amine groups in ionizable lipids get protonated and facilitate the transport of chloride ions to equilibrate the membrane charge and osmotic pressure until the membrane is disrupted and the genetic material is delivered into the cytosol (191). By increasing the degree of unsaturation of the ionizable lipid hydrophobic tail (192) as well as by tuning its pK_a_ (193, 194), it is possible to increase endosomal escape and therefore vaccine potency. Ionizable lipids allow high rates of mRNA encapsulation. Due to the low pH of the aqueous phase during manufacturing, ionizable lipids become positively charged, which promotes the interaction with the negatively charged mRNA backbone. This facilitates the mRNA incorporation into the forming LNP. After LNP formation, a buffer exchange step ensures the buffer goes back to physiological pH. Another component of currently used LNPs is the PEGylated lipid, composed of a hydrophilic PEG polymer conjugated to a hydrophobic lipid anchor. The PEG polymer is situated toward the environment, while the lipid anchor is buried toward the LNP core. The PEGylated lipid increases the LNP circulation time as it prevents opsonin binding and determines particle size during manufacturing, preventing particle fusion. By changing the PEG lipid anchor length, it is possible to tune its shedding rate from the LNP surface, which is essential to promote cellular uptake and endosomal escape. Phospholipids and cholesterol are also employed as contributors to the LNP structural integrity and phase transition behavior. They assist on the mRNA encapsulation and ensure LNP stability over time (190). The versatility of the LNP platform allows for a multitude of modifications in the lipid components and/or proportions to achieve specific characteristics that make them preferentially accumulate in specific organs (195). Moreover, the LNP itself can possess immunogenic properties, which may be reduced to decrease inflammation and deleterious interactions (196–198). As addressed in this review, LNPs can also be functionalized with molecules that specifically interact with cellular receptors to increase their uptake by target cells. This is the concept behind the design of active targeting nanoparticles, which we believe represents the basis for the future of mRNA vaccines.

**Figure 3:**
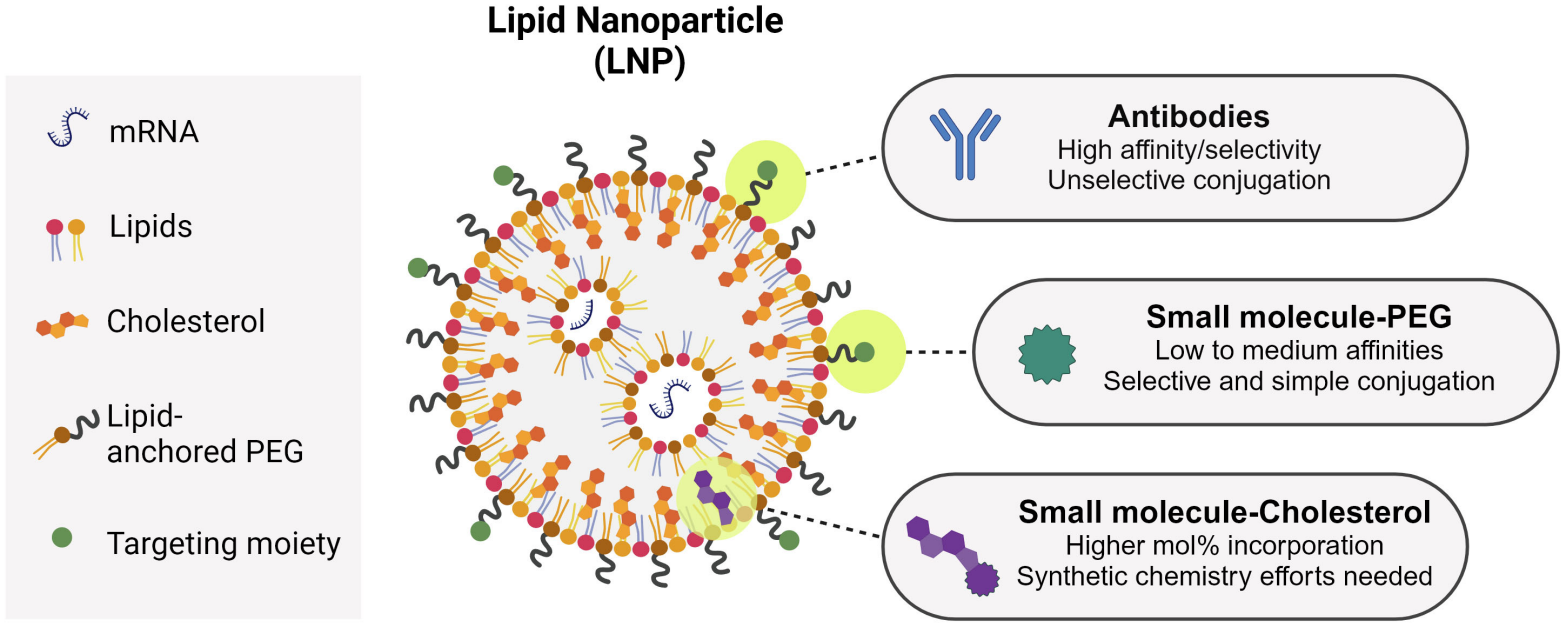
LNP structure, composition, and targeting ligand incorporation strategies.

As seen above, antibodies are bullets of choice to target LNPs toward a specific receptor (**Table 1**). Antibodies are generally conjugated to LNPs after their formulation using PEGylated lipids with functional terminal groups, such as maleimide (199). This process is rather straightforward, but it is difficult to characterize the obtained functionalized particles and to quantify the conjugated antibodies (200). Moreover, chemically conjugating an antibody on the LNP surface does not offer full control of its orientation, and may result in antibodies unable to properly interact with their targets [although this field is making considerable progresses (201)]. Regarding LNP small molecule-functionalization, so far two main strategies have been described: through PEG or through cholesterol (**Figure 3**). Mannosylated PEG has been one of the first examples of sugar-based targeting to deliver GFP DNA to Kupfer cells *in vitro* and *in vivo* (86). PEG has been seen as the ideal candidate to ensure maximum ligand exposure as compared to cholesterol, since its hydrophilic tail is exposed toward the environment, as opposed to cholesterol, which is in the LNP inner structure. With reports demonstrating that PEG gradually sheds from the LNP upon injection (202, 203), PEG might not be the best candidate to ensure LNP targeting after injection. Therefore, other strategies were put in place, with mannosides conjugated to cholesterol instead (204). Indeed, Goswami et al. showed that mannosylated cholesterol improved the internalization and potency of an anti-RSV Self-Amplifying mRNA vaccine (SAM) (176). Recently, some examples of MR-targeted siRNA have also been published, with ligand directly attached to the siRNA or to a siRNA-encapsulating particle (205, 206).

### 4.2 Targeting ligand affinity, the higher the better?

A common approach for targeting (be it of antigens or therapeutic small molecules) relies on the use of antibodies as targeting agents. The resulting construct generally follows a 1:1 stoichiometry in which the antibody is fused to one protein or conjugated to one small molecule. For nucleic acids, the necessity of an encapsulation platform (e.g. an LNP) allows for antibody-guided targeting, but also for the use of smaller targeting ligands like sugars or peptides, multivalently displayed on the particle’s surface. The use of small molecules present advantages over antibodies regarding cost-effectiveness and scalability (**Figure 3**). The use of small molecules comes with the trade-off that the ligand affinity for its target is lower compared to antibodies. This is compensated by the multivalent display of the ligands, that will result in improved avidity (207–209). In the case of sugars, the weak affinities typically observed for monosaccharides can be improved by ligand elongation (210, 211). For example, DC-SIGN binds 130-fold more tightly to an *N*-acetyl-glucosamine-mannose oligosaccharide than to mannose (212). In the case of peptides as targeting ligands, affinities can be improved by optimizing the sequences and/or the peptide’s 3D configuration (213).

CLRs usually display weak affinities toward their carbohydrate ligands, but weakness of the interaction can be overcome by their multimeric nature, i.e. their ability to cluster and/or display multiple binding sites in close proximity to enhance avidity (214, 215). For many receptors, avidity is maximized by multivalent structures (e.g. DC-SIGN is a tetramer, Langerin a trimer, and the MR linearly contains 8 CRDs). Therefore, high-affinity binding to the receptor can be achieved through the interaction of multiple saccharides with multiple CRDs at the same time (216). Avidity is the product of three factors: affinity, valency, and spatial arrangement. To this end, ligand distancing plays a crucial role on multivalent receptor engagement, and can be optimized with physical parameters such as ligand density, linker and ligand length (30, 217–219). Of note, progresses in characterization techniques now allow for the precise quantification of targeting ligand display on a particle surface (220). Interestingly, particle size also influences its receptor-mediated cellular uptake: indeed, Fehres et al. reported that a Lewis Y (LeY)-functionalized liposome lead in DC-SIGN-dependent antigen presentation, while a LeY-bearing peptide was exclusively taken up and presented through Langerin. This exemplifies how the delivery vehicle design and target receptor choice are intertwined.

In conclusion, the multivalent display of smaller ligands can compensate for their weaker affinities in comparison to antibodies. In the specific case of mRNA-vaccine targeting, a strong targeting ligand-receptor interaction as with an antibody can be deleterious for endosomal escape (165) and result in poor antigen translation. This represents a strong drawback to the use of antibodies to target recycling endocytic receptors like Langerin (221). On the other hand, in the case of CLRs, the acidification of the endosome results in a Calcium-dependent affinity drop which disrupts the interaction between the receptor and the targeting ligand and facilitates its escape and/or prevents its recycling (99).

## 5 Discussion

Immune cell-targeted mRNA delivery holds promises to improve vaccine quality and potency. The growing expertise in targeted antigen delivery and the increasing understanding of mRNA formulation need to merge to enable the development of potent DC-targeted mRNA vaccine with improved potency and distribution profile. This may happen only if our knowledge in immunology drives us toward the definition of appropriate targets, and if chemistry and biomolecular technologies enable their specific targeting.

Overall, targeting specific receptors on innate immunity-associated cells could create a stronger and longer-lasting immune response tailored for vaccines applications. Moreover, a combined targeting strategy involving different innate immune cell subsets might be attractive, given their distinct and central role in the immune response. For instance, macrophages drive inflammatory responses, whereas dendritic cells assume a key role in antigen presentation to T cells. Therefore, the simultaneous targeting of both cell types could contribute to the initiation of an improved antigen-specific immune response. In this context, further research will be needed to identify potential common key receptors among different cell types, or to formulate LNPs with a combination of several targeting ligands.

RNA delivery vehicles must be carefully developed, considering the requirements imposed by the complex process of selectively delivering mRNA to a single cell-type. Nanoplatform design should encompass the components’ compatibility in the formulation step, as well as the necessity of maintaining an efficient endosomal escape and an adequate multivalent ligand display. Although the design of such vehicles is complex, and further research is still needed, we are confident about the impact that this technology could have on the deployment of new vaccines worldwide, for both prevalent and emerging diseases.

## Author contributions

BC: Writing – original draft, Writing – review & editing. MD: Conceptualization, Writing – original draft, Writing – review & editing. CS: Conceptualization, Writing – original draft, Writing – review & editing. FS: Writing – original draft, Writing – review & editing. MB: Writing – review & editing. DS: Conceptualization, Writing – review & editing.
